# Different personality factors drive work and non-work creativity

**DOI:** 10.3389/fpsyg.2023.1078874

**Published:** 2023-01-26

**Authors:** Amy Shaw, Qi Yu

**Affiliations:** ^1^Department of Psychology, Faculty of Social Sciences, University of Macau, Taipa, Macau SAR, China; ^2^NUS Business School, National University of Singapore, Singapore, Singapore

**Keywords:** creativity, big 5, personality, everyday creativity, work contexts, non-work settings

## Abstract

This study examined whether creativity at work and outside of work had the same (or different) Big Five personality antecedents. Results revealed that although self-reported personal creativity outside of the workplace was related to the Openness to Experience trait only, supervisor-rated work creativity was associated with Openness, Extraversion, and Conscientiousness, and that after controlling for the effects of Extraversion and Conscientiousness, Openness did not contribute incremental validity in predicting work creativity, whereas when the impact of Openness was controlled for, Extraversion and Conscientiousness incrementally contributed to the prediction. Therefore, the study supported that while creativity was consistently driven by Openness across settings, the predictive effects of other traits (i.e., Extraversion and Conscientiousness) on creativity varied in work and non-work environments. Study limitations and implications for research and practices are discussed.

## Introduction

The study of personality antecedents is one of the long-standing areas in creativity research (Feist, 1998; Sternberg and Lubart, 1999; Batey and Furnham, 2006). Within the Big Five personality structure, Openness to Experience, defined as the disposition to be original, imaginative, intellectually curious, and open to new ideas or experiences (McCrae and Costa, 1989), is undoubtedly the most important trait to creativity—past research has consistently shown that Openness is a positive predictor of creativity across a multitude of measures in diverse domains (King et al., 1996; Feist, 1998; Batey and Furnham, 2006; Silvia et al., 2009; Puryear et al., 2019; Shaw, 2021; Weiss et al., 2021). As for the other four traits, previous studies largely found them to exhibit mixed patterns of relationships with creativity, though some personality traits appeared to matter more in certain domains. For instance, in the seminal meta-analytic review by Feist (1998), Conscientiousness was identified as a trait crucial to scientific creativity, whereas relatively high levels of Neuroticism (or emotional instability and sensitivity) were found to be common among creative artists.

Acknowledging that creativity is domain specific and context dependent (Hennessey and Amabile, 2010; Zeng et al., 2011; Barbot et al., 2019), past studies mostly explored the conceptualization and assessment criteria of the creativity construct in various domains and contexts (e.g., Kaufman et al., 2008; Montag et al., 2012; Piffer, 2012; Simonton, 2012; Acar et al., 2017; Walia, 2019; Shaw, 2022) as well as emphasized the facilitating and/or inhibiting effects of different contextual factors on creativity while often simply regarding the Openness personality trait as an individual’s creative potential, likely for the sake of restricting the scope of the work (e.g., Amabile, 1996; Shalley et al., 2004; Erez and Nouri, 2010; Glăveanu, 2010; Strickland and Towler, 2011; Anderson et al., 2014; also see Rhodes, 1961, for the “creative press” perspective). Not many studies so far have focused on directly examining and comparing the effects of personality factors (especially other traits than Openness) on creativity in different real-life settings, which is somewhat surprising given the long history of personality studies in creativity research (Puryear et al., 2017) and the wide recognition that actual creative behaviors/performance in the real world vary a lot across situations (Hennessey and Amabile, 2010; Beghetto, 2014; Barbot et al., 2019). Given the definitions and criteria of creativity may change from one setting to another and different contextual cues could stimulate or hinder the expression of certain creative behaviors (trait activation theory; Tett and Burnett, 2003), it stands to reason that different sets of personality traits shall be associated with creativity expressed in different contexts (e.g., at school, at work, or in one’s personal life).

In this paper, we focus on the work vs. non-work creativity of working adults. Unlike work creativity that by definition must be novel and useful to the organization (Amabile, 1996; Zhou and George, 2001), non-work creativity in people’s personal lives may not be useful or practically valuable at all and may not even be novel in others’ eyes (Batey, 2007; Richards, 2007; Benedek et al., 2020). According to Richards (2007), at leisure people engage in wide-ranging personally-expressive creative activities (e.g., painting, knitting, woodworking, developing new recipes, writing humorous stories, or making home decorations) of which the ensuing outcomes may or may not be novel and/or useful, and the engagement in such creative acts is meant to be personal and assessed by the individual solely. In other words, the avocational pursuits people take up outside of work could be deemed as creative regardless of social recognition or utility, as long as the activities are new and personally meaningful to the creators themselves. Rather than pursuing any attention or public credibility related to economic and societal contributions, motives for exploring these trivial or even “mundane creative experiences” (Conner et al., 2018, p. 187) include broadening life experiences, developing personal values, learning new things, increasing one’s own knowledge or demonstrating the creative self to oneself (Maslow, 1974), as well as coping with stress and elevating health/well-being in everyday life (Peterson and Seligman, 2004; Richards, 2007; Leckey, 2011; Benedek et al., 2020; Acar et al., 2021). As such, non-work creativity in personal lives may co-occur with work creativity in a formal setting, but these two are quite distinct in terms of the specific behavioral forms, resultant outcomes, motives behind the behaviors, and probably the personality antecedents as well. The present investigation thus aims to make a simple and direct comparison of the Big Five personality predictors of creativity in and outside of the workplace.

## Materials and methods

### Participants and procedure

A total of *N* = 171 management consultants and business analysts and their direct supervisors at a middle-sized consulting company in the United States participated in the study voluntarily. All the employee participants had worked at the company for at least 9 months which ensured the supervisors’ familiarity with their performance on the job.

Participants first signed the consent form online and then proceeded to complete a demographic questionnaire on their age, gender, and ethnicity as well as measures of their personality and creativity in personal life. Supervisors of the employee participants also provided their consent and responded to surveys on each participant’s creativity at work. All the responses from the employee participants and supervisors were confidential and only accessible to the researchers. We had no missing data so that we obtained a final analysis sample of *N* = 171 employee participants (average age = 28.75 [*SD* = 3.39] years old; 53.6% male; 79.1% Caucasian/White) with supervisory assessment on their work creativity.

### Measures

#### Personality

The Big Five personality traits were assessed using the 50-item International Personality Item Pool inventory (IPIP; Goldberg et al., 2006) *via* a 5-point Likert scale (1 = Very inaccurate, 5 = Very accurate). Each trait scale on the IPIP contains 10 items. The employee participants were instructed to describe themselves as they generally were as honestly and accurately as possible in relation to others they knew of the same sex and roughly their same age. In scoring, the average of all item responses on each trait scale was used as the trait scale score. All trait scales had satisfactory estimated reliabilities using the Cronbach’s alpha coefficient: 0.86 (Extraversion), 0.81 (Agreeableness), 0.83 (Conscientiousness), 0.79 (Emotional Stability), and 0.78 (Openness to Experience).

#### Creativity in personal life

The 34-item self-report Biographical Inventory of Creative Behaviors (BICB; Batey, 2007) was used to measure everyday creative activities/behaviors outside of work (i.e., in people’s personal lives and leisure time). The BICB presents a checklist of common creative activities (such as *Drawn a cartoon* or *Made up a joke*) people might have done during the past 12 months using a binary Yes/No response format (which then yielded 1/0 item scores). Participants were instructed to select “Yes” if any activity applies to them in their non-work time and were encouraged to answer as honestly and truthfully as possible. The sum score of all items was calculated to indicate the level of everyday creativity for which the possible score range was 0–34. The BICB scale had satisfactory estimated reliability (Cronbach’s alpha coefficient was 0.81).

#### Creativity at work

Creativity at work was measured using the 13-item work creativity scale developed by Zhou and George (2001) on a 5-point Likert scale (1 = Very inaccurate, 5 = Very accurate). In the work setting, creativity refers to the extent to which an employee contributes ideas, solutions, or products that are both novel and useful to the organization (Zhou and George, 2001). The assessment of work creativity in this sample relied on supervisory ratings. As such, supervisors were instructed to report on their observations of the employee participants’ creative behaviors in the workplace within the past year as honestly and accurately as possible (supervisors were informed that their ratings would be kept confidential and accessible to the researchers only). A sample item states “Suggests new ways of performing work tasks.” The average of all items was taken as the measure of work creativity. The scale exhibited satisfactory estimated reliability (Cronbach’s alpha coefficient was 0.83).

## Analyses and results

Descriptive statistics and Pearson’s correlation results for the main variables are presented in Table 1. As expected, Openness to Experience was positively and moderately related to both work creativity (*r* = 0.30, *p* < 0.001) and non-work everyday creativity (*r* = 0.28, *p* < 0.001). Extraversion and Conscientiousness were also found to be positively related to work creativity with moderate or small to moderate effect sizes (*r* = 0.30, *p* < 0.001, and *r* = 0.26, *p* < 0.001, respectively). In addition, work creativity and non-work daily creativity were positively correlated with each other (*r* = 0.22, *p* < 0.001), and the magnitude of the correlation coefficient was small to moderate. As displayed in Table 1, age and gender were not significantly correlated with any of other study variables and therefore were omitted from subsequent analyses.

**Table 1:** Means, standard deviations, scale reliabilities, and inter-variable correlations.

Variables	*M*	*SD*	1	2	3	4	5	6	7	8
1. Extraversion	3.79	0.56	*(0.86)*							
2. Agreeableness	3.55	0.58	0.20**	*(0.81)*						
3. Conscientiousness	3.62	0.59	0.15	0.19*	*(0.83)*					
4. Emotional Stability	3.60	0.51	−0.16*	−0.13	0.20**	*(0.79)*				
5. Openness to Experience	3.70	0.67	0.18*	0.14	−0.11	−0.08	*(0.78)*			
6. Work Creativity	3.95	0.78	0.30***	−0.10	0.26***	0.07	0.30***	*(0.83)*		
7. Everyday Creativity	9.20	4.81	0.12	0.11	−0.08	−0.06	0.28***	0.22**	*(0.81)*	
8. Age	28.75	3.39	−0.04	0.07	0.05	−0.03	0.06	−0.02	0.06	–
9. Gender (Female = 0, Male = 1)	0.54	–	0.06	−0.03	0.04	0.08	−0.05	0.03	−0.04	−0.01

*N* = 171. Italic values represents Cronbach’s alpha reliabilities are on the diagonal. *p < 0.05; **p < 0.01; ***p < 0.001; two-tailed.

Given that three personality traits (Extraversion and Conscientiousness in addition to Openness to Experience) were all found to be related to work creativity, we further performed a hierarchical regression analysis with work creativity as the dependent variable and the three personality traits as predictor variables entered in two successive steps (Extraversion and Conscientiousness at Step 1 and Openness at Step 2), to examine whether any unique variance in work creativity was explained by Openness (the most robust and well-established personality predictor of creativity) over and above Extraversion and Conscientiousness. As displayed in Table 2, Extraversion and Conscientiousness explained 12% of the variance in work creativity (*R^2^* = 0.12, *p* < 0.01) at Step 1 but at Step 2 after introducing Openness into the regression, we only found a negligible change in the explained variance (4%); a sensitivity analysis revealed that with the present sample size (*N* = 171), we had 80% statistical power to identify a change of Δ*R*^2^ = 0.05 and therefore, the current study was not sensitive enough to detect a small effect of Δ*R*^2^ = 0.04. As such, though the hierarchical regression analysis did not find meaningful incremental predictive effects of Openness above and beyond the effects of Extraversion and Conscientiousness, it might have been a result from a relatively small sample size in the study and thus the findings shall be interpreted with caution.

**Table 2:** Hierarchical regression results for effects of personality predictors on work creativity (extraversion and conscientiousness at Step 1; openness at Step 2).

Predictor	*β*	*sr^2^*
Step 1: *R*^2^ = 0.12**		
Extraversion	0.25**	0.048
Conscientiousness	0.23**	0.036
Step 2: *R*^2^ = 0.16***, Δ*R*^2^ = 0.04		
Openness to experience	0.21*	0.032

*N* = 171. *p < 0.05; **p < 0.01; ***p < 0.001; two-tailed. *sr^2^*, semipartial correlation squared.

We then conducted another hierarchical regression (Table 3) with Openness being entered at Step 1 and Extraversion and Conscientiousness being entered at Step 2 and found that after the impact of Openness was controlled for at Step 1, Extraversion and Conscientiousness accounted for an additional 12% of the variance in the dependent variable work creativity (Δ*R*^2^ = 0.12, *p* < 0.01) at Step 2, indicating that Extraversion and Conscientiousness incrementally contributed to the prediction of work creativity over and above Openness.

**Table 3:** Hierarchical regression results for effects of personality predictors on work creativity (openness at Step 1; extraversion and conscientiousness at Step 2).

Predictor	*β*	*sr^2^*
Step 1: *R*^2^ = 0.04		
Openness to experience	0.21*	0.032
Step 2: *R*^2^ = 0.16***, Δ*R*^2^ = 0.12**		
Extraversion	0.25**	0.048
Conscientiousness	0.23**	0.036

*N* = 171. *p < 0.05; **p < 0.01; ***p < 0.001; two-tailed. *sr^2^*, semipartial correlation squared.

## Discussion

The purpose of this study was to examine and directly compare the Big Five personality predictors of creativity in and outside of the workplace. Contributing to the line of research on personal antecedents of creativity, the study results revealed different sets of Big Five traits linked to creativity exhibited at work versus creativity in one’s personal life. Specifically, we found that although only Openness to Experience contributed to non-work creativity, Openness, Extraversion, and Conscientiousness were all positively related to work creativity and remained significant predictors in the regression results. Moreover, Openness did not show incremental validity in predicting work creativity after controlling for the effects of Extraversion and Conscientiousness, whereas Extraversion and Conscientiousness incrementally contributed to the prediction of work creativity above and beyond Openness.

These results have implications for understanding the role of personality in work and non-work settings. Given the considerable empirical support for the positive association of Openness with creativity in the literature (e.g., Feist, 1998; Puryear et al., 2017, 2019; Weiss et al., 2021; Shaw and Choi, 2023), most of the organizational studies examining employee creativity have traditionally focused on the effects of situational factors in the workplace [e.g., leadership, managerial systems, time deadlines; see Anderson et al. (2014) and Shalley et al. (2004) for detailed reviews of contextual characteristics in the workplace] while restricting the scope of their research by treating Openness as the creative personality trait (e.g., Amabile, 1996; Shalley et al., 2004; Hunter et al., 2007; Erez and Nouri, 2010; Glăveanu, 2010; Strickland and Towler, 2011), which inevitably led to somewhat underappreciation of the impact of other traits on creativity. Our study suggested that although employees’ personal creativity outside of work was associated with Openness solely, their work creativity as rated by supervisors was driven by Extraversion and Conscientiousness in addition to Openness, and that after controlling for the effects of Extraversion and Conscientiousness, Openness did not contribute meaningful incremental prediction of work creativity, but Extraversion and Conscientiousness did explain unique variance in work creativity over Openness.

The revealed associations of Extraversion and Conscientiousness with work creativity in the study are in line with prior research that found positive effects of Extraversion and Conscientiousness on job-related proactivity (Gong et al., 2012; Neal et al., 2012) and job performance in general (Barrick and Mount, 1991; Costa and McCrae, 1992; Goldberg et al., 2006). In organizational settings, while employees with higher levels of Openness may be more dispositioned to embrace novel ideas and come up with new ways of doing things, oftentimes it also requires risk-taking and proactive tendencies (features of Extraversion) to initiate changes that challenge the status quo—the expressive extroverts are, at a minimum, more likely to voice and share their own opinions and suggestions compared to their introvert colleagues, especially when in front of a group of people at work (Costa and McCrae, 1992; Batey et al., 2010). Extraversion also has been associated with creative self-efficacy which contributes to individuals’ creative activities and performance (Karwowski and Lebuda, 2016; Shaw et al., 2021). Similarly, although Conscientiousness is typically not considered a personality predictor of creativity in general and in some cases has even been found to be negatively associated with creativity because of the tendency to obey rules and conform to existing norms (Raja et al., 2004; Batey et al., 2010), the achievement-striving and hardworking characteristics of Conscientiousness are crucial to the development of innovative business plans, new and better problem solutions, or any type of quality product (Howell and Higgins, 1990; Feist, 1998; George and Zhou, 2001). Therefore, one could imagine that there are people high on Openness but low on Extraversion and/or Conscientiousness who might nonetheless be perceived by their supervisors as not quite creative owing to their lack of exhibited actual creative behaviors/performance in the workplace.

That said, rather than discounting the role of Openness, the current study served more as an empirical demonstration that because creativity exists in different forms in distinct contexts such as work vs. non-work environments, one shall consider the personality-creativity relationship within its specific context and further, may try to bridge the gap between creative expression at work and outside of work (Runco, 2007; Runco et al., 2021, 2022; see also Beghetto, 2014 for a discussion of creative suppression and mortification in certain contexts). For instance, working professionals who are high on Openness (but low on Extraversion and/or Conscientiousness) and generally creative in their personal lives, may need to work more on expressing their creativity at work purposefully and diligently so as to translate their creative potential (e.g., divergent thinking capacity, the trait Openness) to actual creative performance and career success; on the other hand, supervisors are also recommended to pay more attention to the creative potential of those introverted employees and encourage the introverts to exhibit creativity more often *via* reward systems.

Note that the findings discussed above shall be read in light of a few important study limitations. First, as with most previous field studies on employee creativity, we used supervisor ratings as the work creativity measure which circumvented self-enhancement bias in self-reports (Dunning et al., 2004), but supervisory performance ratings might suffer from other types of biases including the ubiquitous halo effect (tendency for positive judgment about a person/product in one aspect to positively influence evaluation of the person/product in other areas; Cooper, 1981; Nathan and Tippins, 1990). It is likely that supervisors overrated creative performance for those who might not be really creative but were employees with overall good performance on the job. Given that organizational research has consistently found Extraversion and Conscientiousness to be related to greater supervisor-rated overall job performance (Barrick and Mount, 1991; Goldberg et al., 2006), the present results regarding the predictive effects of Extraversion and Conscientiousness on work creativity, might stem from a general impression of the employee being a good performer at work. Therefore, future research with both work creativity and overall job performance ratings could help to clarify whether the creative dimension ratings add additional variance to that of the overall performance ratings and if so, whether Extraversion and Conscientiousness would still be found to be predictors of work creativity. Second, as previously noted, the null result of incremental validity of Openness in predicting work creativity over Extraversion and Conscientiousness could have resulted from the relatively small sample size in the study. We therefore call for future validation of this finding in a larger sample with adequate power to detect a significant effect (if any), which would give us more confidence to determine whether or not Openness may account for unique and additional variance in work creativity over and above Extraversion and Conscientiousness. Third, our sample was a group of management consultants and business analysts working in the consulting industry, so that the present findings may not generalize to other job types or occupations. For example, in other industries such as information technology or engineering design, it would be interesting to see whether Extraversion (especially the expressiveness aspect) would play an important role in predicting supervisory ratings of creativity all the same. Fourth, it also must be acknowledged that because the current study narrowly focused on the personality-creativity relationship, we did not explore or control for other possible confounding variables such as divergent thinking skills and intelligence. Another drawback of this study is that as with other cross-sectional studies, it lacks a longitudinal design that could allow for observing within-person changes of exhibited creativity over time; no cause-and-effect relationships could be drawn from our study either.

Despite the limitations, the present study added new knowledge about how personality drives creative behaviors/performance in different settings (work vs. non-work environments). The results from this study shall encourage researchers to get more discerned when asserting or interpreting the personality-creativity relationship. Given the critical role of creativity in the human society and the lack of consensus on many issues in the field including the creative gap in different contexts (Anderson et al., 2014; Whorton et al., 2017; Runco et al., 2021, 2022), we call for more comparisons of the personality antecedents of creativity in different situations as well as in-depth examinations of possible interactions between those personal attributes and various work/life conditions.

## Data availability statement

The raw data supporting the conclusions of this article will be made available by the authors, without undue reservation.

## Ethics statement

The studies involving human participants were reviewed and approved by the University of Macau. The patients/participants provided their written informed consent to participate in this study.

## Author contributions

All authors contributed to the conceptualization, data collection, data analysis, and manuscript preparation. All authors contributed to the article and approved the submitted version.

## Funding

This study was supported from the MYRG Research Program of the University of Macau (Reference number: MYRG2022-00274-FSS) is gratefully acknowledged.

## Conflict of interest

The authors declare that the research was conducted in the absence of any commercial or financial relationships that could be construed as a potential conflict of interest.

## Publisher’s note

All claims expressed in this article are solely those of the authors and do not necessarily represent those of their affiliated organizations, or those of the publisher, the editors and the reviewers. Any product that may be evaluated in this article, or claim that may be made by its manufacturer, is not guaranteed or endorsed by the publisher.

## References

[ref1] Acar S. Burnett C. Cabra J. F. (2017). Ingredients of creativity: originality and more. Creat. Res. J. 29, 133–144. doi: 10.1080/10400419.2017.1302776

[ref2] Acar S. Tadik H. Myers D. Van der Sman C. Uysal R. (2021). Creativity and well-being: a meta-analysis. J. Creat. Behav. 55, 738–751. doi: 10.1002/jocb.485

[ref3] Amabile T. M. (1996). Creativity in Context: Update to the Social Psychology of Creativity. Boulder, CO: Westview Press.

[ref5] Anderson N. Potočnik K. Zhou J. (2014). Innovation and creativity in organizations: a state-of-the-science review, prospective commentary, and guiding framework. J. Manag. 40, 1297–1333. doi: 10.1177/0149206314527128

[ref6] Barbot B. Hass R. W. Reiter-Palmon R. (2019). Creativity assessment in psychological research: (re)setting the standards. Psychol. Aesthet. Creat. Arts 13, 233–240. doi: 10.1037/aca0000233

[ref7] Barrick M. R. Mount M. K. (1991). The big five personality dimensions and job performance: a meta-analysis. Pers. Psychol. 44, 1–26. doi: 10.1111/j.1744-6570.1991.tb00688.x

[ref8] Batey M. (2007). A psychometric investigation of everyday creativity. Unpublished Doctoral Thesis. University College, London, United Kingdom.

[ref9] Batey M. Chamorro-Premuzic T. Furnham A. (2010). Individual differences in ideational behavior: can the big five and psychometric intelligence predict creativity scores? Creat. Res. J. 22, 90–97. doi: 10.1080/10400410903579627

[ref10] Batey M. Furnham A. (2006). Creativity, intelligence, and personality: a critical review of the scattered literature. Genet. Soc. Gen. Psychol. Monogr. 132, 355–429. doi: 10.3200/MONO.132.4.355-430, PMID: 18341234

[ref11] Beghetto R. A. (2014). Creative mortification: an initial exploration. Psychol. Aesthet. Creat. Arts 8, 266–276. doi: 10.1037/a0036618

[ref12] Benedek M. Bruckdorfer R. Jauk E. (2020). Motives for creativity: exploring the what and why of everyday creativity. J. Creat. Behav. 54, 610–625. doi: 10.1002/jocb.396

[ref13] Conner T. S. DeYoung C. G. Silvia P. J. (2018). Everyday creative activity as a path to flourishing. J. Posit. Psychol. 13, 181–189. doi: 10.1080/17439760.2016.1257049

[ref14] Cooper W. H. (1981). Ubiquitous halo. Psychol. Bull. 90, 218–244. doi: 10.1037/0033-2909.90.2.218

[ref15] Costa P. T. McCrae R. R. (1992). Neo PI-R Professional Manual. Odessa, FL: Psychological Assessment Resources.

[ref16] Dunning D. Heath C. Suls J. M. (2004). Flawed self-assessment: implications for health, education, and the workplace. Psychol. Sci. Public Interest 5, 69–106. doi: 10.1111/j.1529-1006.2004.00018.x 26158995

[ref17] Erez M. Nouri R. (2010). Creativity: the influence of cultural, social, and work contexts. Manag. Organ. Rev. 6, 351–370. doi: 10.1111/j.1740-8784.2010.00191.x

[ref18] Feist G. J. (1998). A meta-analysis of personality in scientific and artistic creativity. Personal. Soc. Psychol. Rev. 2, 290–309. doi: 10.1207/s15327957pspr0204_5, PMID: 15647135

[ref19] George J. M. Zhou J. (2001). When openness to experience and conscientiousness are related to creative behavior: an interactional approach. J. Appl. Psychol. 86, 513–524. doi: 10.1037/0021-9010.86.3.513, PMID: 11419810

[ref20] Glăveanu V. P. (2010). Creativity in context: the ecology of creativity evaluations and practices in an artistic craft. Psychol. Stud. 55, 339–350. doi: 10.1007/s12646-010-0056-8

[ref21] Goldberg L. R. Johnson J. A. Eber H. W. Hogan R. Ashton M. C. Cloninger C. R.. (2006). The international personality item pool and the future of public- domain personality assessment. J. Res. Pers. 40, 84–96. doi: 10.1016/j.jrp.2005.08.007

[ref22] Gong Y. Cheung S. Y. Wang M. Huang J. C. (2012). Unfolding the proactive process for creativity: integration of the employee proactivity, information exchange, and psychological safety perspectives. J. Manag. 38, 1611–1633. doi: 10.1177/0149206310380250

[ref23] Hennessey B. A. Amabile T. M. (2010). Creativity. Annu. Rev. Psychol. 61, 569–598. doi: 10.1146/annurev.psych.093008.100416 19575609

[ref24] Howell J. M. Higgins C. A. (1990). Champions of technological innovation. Adm. Sci. Q. 35, 317–341. doi: 10.2307/2393393

[ref25] Hunter S. T. Bedell K. E. Mumford M. D. (2007). Climate for creativity: a quantitative review. Creat. Res. J. 19, 69–90. doi: 10.1080/10400410709336883

[ref26] Karwowski M. Lebuda I. (2016). The big five, the huge two, and creative self-beliefs: a meta-analysis. Psychol. Aesthet. Creat. Arts 10, 214–232. doi: 10.1037/aca0000035

[ref27] Kaufman J. C. Plucker J. A. Baer J. (2008). Essentials of Creativity Assessment. New York, NY: Wiley.

[ref28] King L. A. Walker L. M. Broyles S. J. (1996). Creativity and the five-factor model. J. Res. Pers. 30, 189–203. doi: 10.1006/jrpe.1996.0013

[ref29] Leckey J. (2011). The therapeutic effectiveness of creative activities on mental well-being: a systematic review of the literature. J. Psychiatr. Ment. Health Nurs. 18, 501–509. doi: 10.1111/j.1365-2850.2011.01693.x, PMID: 21749556

[ref30] Maslow A. H. (1974). “Creativity in self-actualizing people. Readings in human development: a humanistic approach” in Readings in Human Development: A Humanistic Approach. ed. Covin T. M. (New York, NY: MSS Information Corporation), 107–117.

[ref31] McCrae R. R. Costa P. T. (1989). The structure of interpersonal traits: Wiggins’s circumplex and the five-factor model. J. Pers. Soc. Psychol. 56, 586–595. doi: 10.1037/0022-3514.56.4.586, PMID: 2709308

[ref32] Montag T. Maertz C. P. Jr. Baer M. (2012). A critical analysis of the workplace creativity criterion space. J. Manag. 38, 1362–1386. doi: 10.1177/0149206312441835

[ref33] Nathan B. R. Tippins N. (1990). The consequences of halo “error” in performance ratings: a field study of the moderating effect of halo on test validation results. J. Appl. Psychol. 75, 290–296. doi: 10.1037/0021-9010.75.3.290

[ref34] Neal A. Yeo G. Koy A. Xiao T. (2012). Predicting the form and direction of work role performance from the big 5 model of personality traits. J. Organ. Behav. 33, 175–192. doi: 10.1002/job.742

[ref35] Peterson C. Seligman M. E. (2004). Character Strengths and Virtues: A Handbook and Classification. Vol. 1. New York, NY: Oxford University Press.

[ref36] Piffer D. (2012). Can creativity be measured? An attempt to clarify the notion of creativity and general directions for future research. Think. Skills Creat. 7, 258–264. doi: 10.1016/j.tsc.2012.04.009

[ref37] Puryear J. S. Kettler T. Rinn A. N. (2017). Relationships of personality to differential conceptions of creativity: a systematic review. Psychol. Aesthet. Creat. Arts 11, 59–68. doi: 10.1037/aca0000079

[ref38] Puryear J. S. Kettler T. Rinn A. N. (2019). Relating personality and creativity: considering what and how we measure. J. Creat. Behav. 53, 232–245. doi: 10.1002/jocb.174

[ref39] Raja U. Johns G. Ntalianis F. (2004). The impact of personality on psychological contracts. Acad. Manag. J. 47, 350–367. doi: 10.2307/20159586

[ref02] Rhodes M. (1961). An analysis of creativity. The Phi Delta Kappan 42, 305–310.

[ref41] Richards R. (2007). “Everyday creativity: our hidden potential” in Everyday Creativity and New Views of Human Nature: Psychological, Social, and Spiritual Perspectives. ed. Richards R. (Washington, DC: American Psychological Association), 25–53.

[ref42] Runco M. A. (2007). A hierarchical framework for the study of creativity. New Horiz. Educat. 55, 1–9.

[ref43] Runco M. A. Abdulla Alabbasi A. M. Acar S. Ayoub A. E. A. (2022). Creative potential is differentially expressed in school, at home, and the natural environment. Creat. Res. J., 1–8. doi: 10.1080/10400419.2022.2031437

[ref44] Runco M. Shepard A. Tadik H. (2021). How much creative potential is expressed at work? J. Creativ. 32:100016. doi: 10.1016/j.yjoc.2021.100016

[ref45] Shalley C. E. Zhou J. Oldham G. R. (2004). The effects of personal and contextual characteristics on creativity: where should we go from here? J. Manag. 30, 933–958. doi: 10.1016/j.jm.2004.06.007

[ref46] Shaw A. (2021). It works… but can we make it easier? A comparison of three subjective scoring indexes in the assessment of divergent thinking. Think. Skills Creat. 40:100789. doi: 10.1016/j.tsc.2021.100789

[ref47] Shaw A. (2022). Creative Minecrafters: cognitive and personality determinants of creativity, novelty, and usefulness in Minecraft. Psychol. Aesthet. Creat. Arts. doi: 10.1037/aca0000456

[ref01] Shaw A. Choi J. (2023). Get creative to get ahead? How personality contributes to creative performance and perceptions by supervisors at work. Acta Psychologica 233:103835. doi: 10.1016/j.actpsy.2023.103835 36640560

[ref48] Shaw A. Kapnek M. Morelli N. A. (2021). Measuring creative self-efficacy: an item response theory analysis of the creative self-efficacy (CSE) scale. Front. Psychol. 12:2577. doi: 10.3389/fpsyg.2021.678033 PMC8297998 34305732

[ref49] Silvia P. J. Nusbaum E. C. Berg C. Martin C. O’Connor A. (2009). Openness to experience, plasticity, and creativity: exploring lower-order, high-order, and interactive effects. J. Res. Pers. 43, 1087–1090. doi: 10.1016/j.jrp.2009.04.015

[ref50] Simonton D. K. (2012). Taking the U.S. patent office criteria seriously: a quantitative three-criterion creativity definition and its implications. Creat. Res. J. 24, 97–106. doi: 10.1080/10400419.2012.676974

[ref52] Sternberg R. J. Lubart T. I. (1999). “The concept of creativity: prospects and paradigms” in Handbook of Creativity. ed. Sternberg R. J. (New York, NY: Cambridge University Press), 3–16.

[ref53] Strickland S. Towler A. (2011). Correlates of creative behaviour: the role of leadership and personal factors. Can. J. Adm. Sci. 28, 41–51. doi: 10.1002/cjas.157

[ref54] Tett R. P. Burnett D. D. (2003). A personality trait-based interactionist model of job performance. J. Appl. Psychol. 88, 500–517. doi: 10.1037/0021-9010.88.3.500, PMID: 12814298

[ref55] Walia C. (2019). A dynamic definition of creativity. Creat. Res. J. 31, 237–247. doi: 10.1080/10400419.2019.1641787

[ref56] Weiss S. Steger D. Kaur Y. Hildebrandt A. Schroeders U. Wilhelm O. (2021). On the trail of creativity: dimensionality of divergent thinking and its relation with cognitive abilities, personality, and insight. Eur. J. Personal. 35, 291–314. doi: 10.1002/per.2288

[ref57] Whorton R. Casillas A. Oswald F. L. Shaw A. (2017). “Critical skills for the 21st century workforce” in Building Better Students: Preparation for the Workforce. eds. Burrus J. Mattern K. D. Naemi B. Roberts R. D. (New York, NY: Oxford University Press), 47–72.

[ref58] Zeng L. Proctor R. W. Salvendy G. (2011). Can traditional divergent thinking tests be trusted in measuring and predicting real-world creativity? Creat. Res. J. 23, 24–37. doi: 10.1080/10400419.2011.545713

[ref59] Zhou J. George J. M. (2001). When job dissatisfaction leads to creativity: encouraging the expression of voice. Acad. Manag. J. 44, 682–696. doi: 10.2307/3069410

